# Development of a disaster nursing training program for undergraduate interns in mobile cabin hospital settings

**DOI:** 10.3389/fpubh.2025.1672455

**Published:** 2025-09-24

**Authors:** Lan Shi, Min Liu, Gang-Ren Jian, Ji-Min Chen, Yan-Ran Li, Shuang Jin

**Affiliations:** ^1^Shengli Clinical Medical College of Fujian Medical University, Fuzhou, Fujian, China; ^2^Department of Intensive Care Medicine, Fujian Provincial Hospital, Fuzhou University Affiliated Provincial Hospital, Fuzhou, Fujian, China; ^3^Department of Nursing, Fujian Provincial Hospital, Fuzhou University Affiliated Provincial Hospital, Fuzhou, Fujian, China; ^4^School of Nursing, Fujian Medical University, Fuzhou, Fujian, China; ^5^Department of Emergency, Fujian Provincial Hospital, Fuzhou University Affiliated Provincial Hospital, Fuzhou, Fujian, China; ^6^Department of Emergency Intensive Care Medicine, Fujian Provincial Hospital, Fuzhou University Affiliated Provincial Hospital, Fuzhou, Fujian, China; ^7^State University of New York at Buffalo, Buffalo, NY, United States

**Keywords:** Delphi method, disaster nursing, mobile cabin hospital, practical training program, undergraduate nursing interns

## Abstract

**Objective:**

To develop a structured “Disaster Nursing Practice Week” training program for undergraduate nursing interns, tailored to the context of mobile cabin hospitals, with the aim of enhancing disaster nursing competencies and providing a theoretical foundation for practical training.

**Methods:**

The program was designed using the disaster management continuum theory in conjunction with the 2019 International Council of Nurses (ICN) updated *Disaster Nursing Core Competencies* framework. The Delphi method was employed to conduct two rounds of expert consultation, involving 15 professionals with expertise in nursing education, nursing management, disaster nursing, and emergency medicine.

**Results:**

The response rate for both Delphi rounds was 100%. The expert authority coefficient was calculated at 0.93. The coefficient of variation ranged from 0.000 to 0.205 in the first round and from 0.000 to 0.125 in the second round. Kendall’s coefficient of concordance (W) was 0.252 and 0.300, respectively (*p* < 0.001). The finalized training program comprised 8 primary indicators, 14 secondary indicators, and 47 tertiary indicators.

**Conclusion:**

The training program demonstrated a high degree of relevance and alignment with the practical needs of undergraduate nursing interns. It provides a systematic framework designed to enhance clinical competencies and strengthen disaster nursing preparedness within mobile cabin hospital environments.

## 1 Introduction

Globally, numerous unpredictable natural disasters, human-made accidents, and public health emergencies occur annually, posing complex challenges to society. These events not only threaten public health but also compromise the stability of economic and social systems and the sustainability of ecological environments (1–4). As a country with frequent disaster occurrences, China faces unique and urgent challenges in the domain of disaster management (1, 5, 6). Healthcare professionals serve as indispensable components of disaster response teams (7). Among them, nurses represent the largest group and play critical, non-substitutable roles across the full spectrum of disaster management, including mitigation, preparedness, emergency response, and post-disaster recovery phases (1, 2, 7–10). The level of disaster nursing competency directly affects the effectiveness and efficiency of relief efforts undertaken by the broader disaster response teams (11). In addition to qualified nursing professionals, undergraduate nursing students constitute an essential part of the nursing workforce and serve as important human resources for health service delivery in disaster settings. In the event of a disaster, nursing students may be mobilized to support licensed nurses in relief efforts, thereby reinforcing the overall capacity of the healthcare system (12–14). Evidence indicates that nursing students, though not yet licensed, have acquired fundamental clinical skills through formal education and are therefore capable of contributing positively to disaster response efforts (7). During the Coronavirus Disease 2019 (COVID-19) pandemic, numerous nursing graduates entered clinical settings, where they provided critical assistance to patients and helped alleviate workforce shortages (12, 15). These findings highlight the importance of fostering disaster response competencies among undergraduate nursing students.

Currently, in the field of disaster medicine/nursing education, several countries have established influential educational models. For example, the National Disaster Life Support (NDLS) curriculum in the United States focuses on standardized response protocols, while the Emergency Preparedness and Response (EPR) framework in the UK emphasizes command systems and multi-agency collaboration. These models provide important theoretical foundations and a global perspective for this study. Globally, disaster nursing education and training programs for nursing students have demonstrated gradual growth, although significant regional disparities remain (16). Most existing programs prioritize disaster preparedness and emergency response, often emphasizing specific operational skills such as triage and psychological first aid, while underrepresenting comprehensive training in all phases of disaster management—namely, mitigation, preparedness, response, and recovery (4, 10). As a result, current educational interventions often fall short of meeting international standards for disaster preparedness (17). In China, disaster nursing education was initiated relatively late, and traditional training models also face numerous challenges. Including inconsistent curricula and variability in syllabi across academic institutions (18, 19). Furthermore, most programs remain confined to classroom-based learning, with limited integration of disaster nursing practice during clinical internships. The suddenness and irreproducibility of disasters result in the current disaster care training courses having low scene authenticity. There is a critical need to provide structured educational programs that equip nursing students and professionals with both theoretical knowledge and practical competencies in disaster nursing. The most recent edition of the International Council of Nurses (ICN) *Disaster Nursing Core Competencies V2.0* underscores the necessity of delivering multilevel training across all phases of disaster management (9).

Mobile cabin hospitals are composed of interconnected modular units, such as vehicles, tents, and cabin units, equipped to deliver a range of medical services including emergency treatment, surgery, radiologic imaging, laboratory diagnostics, and life support. These modular units can be assembled into enclosed, self-contained zones that can function independently of external environmental conditions, enabling medical teams to provide a full range of clinical services (20). The modular configuration allows for flexible assembly into field hospitals of various sizes, depending on the demands of specific disaster scenarios.

Owing to their high mobility, rapid deployment capabilities, and adaptability to diverse environments, mobile cabin hospitals have become essential in emergency medical response and have played a significant role in China’s earthquake relief efforts and responses to public health emergencies (21). The current study aimed to construct a week-long clinical training program in disaster nursing, termed the “Disaster Nursing Practice Week,” specifically designed for undergraduate nursing interns. This program was developed within the context of mobile cabin hospital deployment and was guided by the ICN Disaster Nursing Core Competencies framework (1, 9). The objective of the program is to enhance nursing interns’ disaster nursing competencies, increase their ability to assess and respond effectively in disaster contexts, promote willingness to participate in disaster relief efforts, and contribute to the development of a resilient and skilled disaster nursing workforce.

## 2 Participants and methods

### 2.1 Participants

#### 2.1.1 Research team establishment

The research team comprised seven members, including three associate chief nurses, two charge nurses, and two staff nurses. Their responsibilities included designing the expert consultation questionnaires, selecting appropriate consultation experts, and conducting statistical analyses of the consultation results.

#### 2.1.2 Selection of consultation experts

The selection of experts is a critical component of the rigor and credibility of the Delphi consultation process, as the representativeness and authority of the panel directly affect the scientific validity and reliability of the findings. It is generally recommended that 10 to 50 experts be selected to ensure representativeness with practical feasibility (11). Panels with fewer participants may lack diversity and diminish the authority of the results, while larger panels may increase logistical complexity and data processing demands. Based on the specific needs of this study, the following inclusion criteria were applied for the selection of expert participants: (1) Voluntary participation, with a high degree of motivation and ability to engage in both rounds of consultation; (2) Possession of a bachelor’s degree or higher and a professional title at the intermediate level or above; (3) A minimum of 10 years of relevant work experience; (4) Previous experience in disaster relief or recognized expertise in the field of disaster nursing; and (5) Experience in disaster nursing education or research, familiarity with disaster nursing training program development, or service on disaster relief teams, professional disaster nursing committees, or as nursing managers in critical care, rehabilitation, or related specialties.

### 2.2 Methods

#### 2.2.1 Preliminary construction of the mobile cabin hospital “disaster nursing practice week” training program

The initial development of the training program—hereinafter referred to as the “training program”—was guided by the disaster management continuum theory and the 2019 ICN *Disaster Nursing Core Competencies*, which outline eight core dimensions relevant to nursing practice in disaster contexts. Through systematic literature review, practical experience of disaster relief in cabin hospitals, and preliminary research, the relevant components of nursing intern training plan in mobile makeshift hospital environment were preliminarily determined. Following iterative internal discussions among research team members, a preliminary version of the training program was constructed, which included eight primary indicators, 13 secondary indicators, and 42 tertiary indicators.

#### 2.2.2 Expert consultation process for training program development

The draft training program was incorporated into the first-round expert consultation questionnaire. The questionnaire comprised four sections: (1) An introductory section outlining the research background, objectives, and methodology; (2) A Delphi survey section, wherein experts were asked to assess the importance of each indicator using a 5-point Likert scale: “very important,” “important,” “moderately important,” “not very important,” and “not important,” corresponding to scores from 5 to 1. Experts were also invited to indicate additions, deletions, or revisions to the listed indicators; (3) A section assessing the authority of participating experts, which included a self-assessment of familiarity with the subject matter and the criteria used evaluating the indicators; and (4) A demographic and professional profile section, collecting information such as education, disaster relief and training experience, and other relevant professional qualifications.

#### 2.2.3 Distribution of Delphi questionnaires

Two rounds of expert consultation were conducted between October 2023 and March 2024. Prior to distributing the questionnaires, brief telephone communications were initiated with selected experts to introduce the study background, objectives, and methodology. Upon obtaining informed consent, the Delphi questionnaires were distributed via email with instructions for return within 2 weeks. A one-month interval was maintained between the two rounds of consultation.

The framework underwent validation through two rounds of Delphi expert consultations, during which items were scored for their importance while incorporating expert revisions and new suggestions. Its reliability and validity were quantitatively assessed using statistical indicators including expert authority coefficients, coefficient of variation, and Kendall’s W-coefficient of concordance. Finally, a standardized process was implemented with multiple rounds of revisions until all domain items met the consistency threshold, after which the final indicator system was aligned with the ICN competency framework. This process ensured the reproducibility of training program design and demonstrated broader potential for promotion and application.

#### 2.2.4 Statistical analysis

All completed questionnaires were coded and subjected to data verification. Double data entry was performed using Excel 2020, and statistical analysis was conducted using SPSS version 26.0. The statistical indicators included: (1) Descriptive statistics for expert demographic characteristics (frequency, composition ratio, mean ± standard deviation); (2) Response rate, calculated as the proportion of valid questionnaires returned; (3) Indicators of opinion concentration, including mean, standard deviation, full score ratio, and selection rate; (4) Measures of agreement among expert responses, assessed using the coefficient of variation and Kendall’s W coefficient; and (5) Assessment of expert authority, reported through the authority coefficient. A significance threshold of *p* < 0.05 was applied.

#### 2.2.5 Quality control

To ensure the scientific validity, reliability, and feasibility of the consultation process, quality control measures were implemented at each stage. These included: (1) Applying strict inclusion and exclusion criteria and inviting a multidisciplinary panel to ensure broad representation; (2) Obtaining informed consent through direct communication following expert selection; (3) Designing Delphi questionnaires using concise and precise language; (4) Sending timely reminders to experts after distribution to maximize the questionnaire return rate; and (5) Including revised results from the first consultation round into the second-round questionnaire, allowing experts to review changes and provide updated evaluations.

## 3 Results

### 3.1 General characteristics of experts

A total of 15 experts participated in and completed both rounds of the Delphi consultation. The panel comprised professionals from four regions in China, Beijing, Fujian, Sichuan, and Yunnan, and included representation from the fields of nursing education, nursing management, disaster nursing, and emergency medicine. Among them, 3 were male and 12 were female. Their ages ranged from 35 to 56 years (mean = 44.07, SD = 6.58), and their work experience ranged from 10 to 38 years (mean = 23.00, SD = 8.50). Regarding educational background, 3 held doctoral degrees, 11 held master’s degrees, and 1 held a bachelor’s degree. In terms of professional titles, 5 were at the intermediate level, 7 at the associate senior level, and 3 at the senior level. A total of 11 experts had prior experience in disaster relief. The general characteristics of the consultation experts are presented in Table 1.

**Table 1:** Demographic and professional characteristics of consultation experts.

Item		Number of cases (*n*)	Composition ratio (%)
Sex	Male	3	20.00
	Female	12	80.00
Age	<40 years old	3	20.00
	40–49 years old	8	53.33
	≥50 years old	4	26.67
Years of work experience	<15 years	2	13.33
	15–19 years	5	33.33
	20–29 years	3	20.00
	≥30 years	5	33.33
Educational attainment	Doctoral degrees	3	20.00
	Master’s degree	11	73.33
	Bachelor’s degree	1	6.67
Professional title	Intermediate-level	5	33.33
	Associate senior-level	7	46.67
	Senior-level	3	20.00
Disaster relief experience	Yes	11	73.33
	No	4	26.67
Professional field	Nursing education	3	20.00
	Nursing management	8	53.33
	Disaster nursing	3	20.00
	Emergency medicine	1	6.67
Institution	University	3	20.00
	University-affiliated hospital	10	66.67
	Local hospital	2	13.33

### 3.2 Expert response rate

The response rate for both rounds of expert consultation was 100%. In the first round, 9 experts (60%) provided a total of 37 comments. In the second round, 5 experts (33%) submitted 8 comments. These response patterns reflect strong engagement and enthusiasm among the experts for participation in the study.

### 3.3 Expert authority level

The authority of the expert panel was evaluated using two components: the judgment basis coefficient and the familiarity coefficient. The judgment basis coefficient was 0.930, and the familiarity coefficient was 0.880. The overall authority coefficient was 0.905. These values indicate a high level of expertise among the participants, supporting the reliability of the consultation outcomes.

### 3.4 Expert opinion coordination level

The coefficient of variation for expert opinions ranged from 0.000 to 0.205 in the first round and from 0.000 to 0.125 in the second round. The decrease in the coefficient of variation during the second round indicates improved convergence of expert opinions. Kendall’s coefficient of concordance (W) was 0.252 in the first round and 0.300 in the second round. Both coefficients were statistically significant (*p* < 0.001). These findings demonstrate meaningful level of agreement among the experts. Detailed results for coordination levels are presented in Table 2.

**Table 2:** Coordination of expert opinions: coefficient of variation and Kendall’s W.

Consultation round	Number of indicators	Coefficient of variation range	Kendall’s W value	χ^2^	*p* value
First round	42	0.000–0.205	0.252	148.122	0.000
Second round	47	0.000–0.125	0.300	197.640	0.000

### 3.5 Expert opinion concentration level

Following two rounds of Delphi consultation, the finalized training program included 8 primary indicators, 14 secondary indicators, and 47 tertiary indicators. The mean importance score for all indicators ranged from 4.80 to 5.00. The standard deviation ranged from 0 to 0.52. The full score rate varied between 73.33 and 100%, while the selection rate ranged from 93.33 to 100%. The coefficient of variation for all indicators was between 0 and 0.125. These metrics reflect a high level of consensus among the experts. The results of the second-round expert consultation on the training program are presented in Table 3.

**Table 3:** Summary of second-round expert consultation results for training program indicators.

Indicators	Mean ± standard deviation	Full score rate (%)	Coefficient of variation
1 Preparation and planning	5.00 ± 0.00	100.00	0.000
1.1 Disaster-related knowledge preparation	4.93 ± 0.26	93.33	0.052
1.1.1 Basic disaster knowledge (definitions, characteristics, classification of causative factors)	4.87 ± 0.35	86.67	0.072
1.1.2 Global common disasters and their impact	4.80 ± 0.56	86.67	0.117
1.1.3 Disaster management principles (PPRR theory)	4.87 ± 0.35	86.67	0.072
1.1.4 Disaster risk assessment	4.93 ± 0.26	93.33	0.052
1.1.5 Current status of disaster nursing in China (nurses’ roles and responsibilities)	4.87 ± 0.52	93.33	0.106
1.2 Pre-disaster relief preparation	4.93 ± 0.26	93.33	0.052
1.2.1 Physical and mental preparation	5.00 ± 0.00	100.00	0.000
1.2.2 Information preparedness (early warnings, contact information of disaster treatment departments at all levels)	4.93 ± 0.26	93.33	0.052
1.2.3 Personal material preparation and equipment use	4.93 ± 0.26	93.33	0.052
1.2.4 Experience sharing session (material preparedness)	4.80 ± 0.41	80.00	0.086
1.3 Pre-construction preparation for mobile cabin hospitals	4.93 ± 0.26	93.33	0.052
1.3.1 EMT construction standards	4.73 ± 0.46	73.33	0.097
1.3.2 Overview of mobile cabin hospital construction (operation mode, personnel and material allocation)	4.80 ± 0.41	80.00	0.086
1.3.3 Mobile cabin hospital assembly	4.73 ± 0.59	80.00	0.125
2 Assessment	5.00 ± 0.00	100.00	0.000
2.1 Pre-relief assessment at disaster scene	5.00 ± 0.00	100.00	0.000
2.1.1 Scene assessment principles	5.00 ± 0.00	100.00	0.000
2.1.2 Relief personnel assessment	5.00 ± 0.00	100.00	0.000
2.1.3 Environmental hazard assessment	5.00 ± 0.00	100.00	0.000
2.2 Health assessment at disaster scene and mobile cabin hospital	4.93 ± 0.26	93.33	0.05
2.2.1 Simple triage and rapid treatment (START) method	5.00 ± 0.00	100.00	0.000
2.2.2 Rapid trauma assessment (head-to-toe)	5.00 ± 0.00	100.00	0.000
2.2.3 In-hospital patient assessment	5.00 ± 0.00	100.00	0.000
3 Intervention	5.00 ± 0.00	100.00	0.000
3.1 Common disaster scene medical skills	5.00 ± 0.00	100.00	0.000
3.1.1 Trauma care (hemostasis, bandaging, immobilization)	5.00 ± 0.00	100.00	0.000
3.1.2 Casualty transport and transfer	5.00 ± 0.00	100.00	0.000
3.1.3 Cardiopulmonary resuscitation	4.87 ± 0.52	93.33	0.106
3.1.4 Other skills (self-rescue tool making and use)	4.80 ± 0.41	80.00	0.086
3.2 Disaster scene response	5.00 ± 0.00	100.00	0.000
3.2.1 Medical response principles	4.93 ± 0.26	93.33	0.052
3.2.2 Public health response principles	4.87 ± 0.35	86.67	0.072
4 Safety assurance	5.00 ± 0.00	100.00	0.000
4.1 Infectious disease protection	5.00 ± 0.00	100.00	0.000
4.1.1 Prevention and control knowledge	4.93 ± 0.26	93.33	0.052
4.1.2 Personal protective equipment types and usage	5.00 ± 0.00	100.00	0.000
4.1.3 Donning/doffing Level II personal protective equipment	5.00 ± 0.00	100.00	0.000
4.1.4 Experience sharing session (personal protection equipment use)	4.93 ± 0.26	93.33	0.052
4.2 Mobile cabin hospital safety assurance	5.00 ± 0.00	100.00	0.000
4.2.1 Facility risk assessment and management (familiarity with emergency shelters, evacuation routes)	4.93 ± 0.26	93.33	0.052
4.2.2 Equipment and drug safety management (oxygen generator and other high-powered equipment, flammable and explosive materials, and drugs)	4.93 ± 0.26	93.33	0.052
4.2.3 Special population risk assessment and management (chronic diseases, mental diseases, disabled persons, etc.)	4.93 ± 0.26	93.33	0.052
5 Recovery	5.00 ± 0.00	100.00	0.000
5.1 Post-disaster psychological relief	5.00 ± 0.00	100.00	0.000
5.1.1 Psychological responses and coping	4.93 ± 0.26	93.33	0.052
5.1.2 Rapid psychological assessment	4.93 ± 0.26	93.33	0.052
5.1.3 Strategies for vulnerable populations (older adults, children, pregnant and postpartum women, bereaved individuals, disabled individuals, etc.)	4.93 ± 0.26	93.33	0.052
5.1.4 Self-relaxation techniques	4.93 ± 0.26	93.33	0.052
5.2 Post-disaster health education	4.93 ± 0.26	93.33	0.052
5.2.1 Early rehabilitation nursing	4.80 ± 0.56	86.67	0.117
5.2.2 Infectious disease prevention and control (health education)	4.93 ± 0.26	93.33	0.052
6 Communication	5.00 ± 0.00	100.00	0.000
6.1 Effective communication skills in disaster relief	5.00 ± 0.00	100.00	0.000
6.1.1 Communication among responders	4.93 ± 0.26	93.33	0.052
6.1.2 Communication with patients	4.93 ± 0.26	93.33	0.052
6.1.3 Use of communication tools	4.93 ± 0.26	93.33	0.052
7 Legal and ethics	5.00 ± 0.00	100.00	0.000
7.1 Disaster-related legal and ethical issues	4.80 ± 0.41	80.00	0.086
7.1.1 Legal and ethical frameworks relevant to disaster nursing	4.80 ± 0.41	80.00	0.086
7.1.2 Ethical considerations in disaster care	4.87 ± 0.35	86.67	0.072
8 Disaster management	5.00 ± 0.00	100.00	0.000
8.1 Mobile cabin hospital emergency care management	4.93 ± 0.26	93.33	0.052
8.1.1 Emergency plan formulation	4.93 ± 0.26	93.33	0.052
8.1.2 Personnel and supply management	4.93 ± 0.26	93.33	0.052
8.1.3 Mass casualty management	4.93 ± 0.26	93.33	0.052
8.1.4 In-hospital patient care coordination	4.87 ± 0.35	86.67	0.072
8.1.5 Rehabilitation guidance and health education	4.80 ± 0.56	86.67	0.117

### 3.6 Consultation results for training hours and teaching methods

Experts proposed revisions to the allocation of instructional time, particularly within the modules addressing preparation and planning, assessment, and disaster management. Following comprehensive discussion, the majority of expert recommendations were incorporated into the final training plan. The finalized training schedule included a total of 35 class hours, with 7 class hours allocated per day. While the instructional duration of each module remained unchanged, additional time for reflection and discussion was included in the implementation plan. We mapped the training items to the ICN Framework of Disaster Nursing Competencies (2019). Among these, Level I competencies such as “fundamental disaster knowledge,” “basic triage skills,” and “personal protection” were all encompassed in this program. Certain Level II competencies, including “interdisciplinary team coordination” and “overall operation management of mobile cabin hospitals,” were also addressed through secondary indicators within Module 8 (Disaster Management). In terms of instructional methods, a multi-modal approach was adopted, incorporating several complementary teaching strategies tailored to the structure of the training program. Details regarding the allocation of training hours and teaching methods are presented in Table 4. In addition, regarding the teaching methods, a multimodal teaching approach combining several optimal instructional methods is adopted in accordance with the training program. As for the selection of teaching faculty, all teaching *Homo sapiens* possess extensive rescue experience, clinical and academic teaching experience, and are members of the National Emergency Rescue Team (Fujian Province).

**Table 4:** Finalized training hours and instructional methods based on expert consultation.

Teaching content		Teaching methods	Class hours
1. Preparation and planning	1.1 Disaster-related knowledge preparation	Theoretical instruction, video demonstration, game interaction, group discussion	2
	1.2 Pre-disaster relief preparation	Theoretical instruction, physical object display, discussion and reflection	2
	1.3 Pre-construction preparation for mobile cabin hospital	Theoretical instruction, video demonstration, on-site construction	3
2. Assessment	2.1 Pre-relief assessment at disaster scene	Theoretical instruction, scenario simulation, group practice	4
	2.2 Health assessment at disaster scene and mobile cabin hospital		
3. Intervention	3.1 Common disaster scene medical skills	Workshop, on-site demonstration, group practice	4
	3.2 Disaster scene response	Theoretical instruction, video demonstration, on-site demonstration	3
4. Safety assurance	4.1 Infectious disease safety protection	Theoretical instruction, video demonstration, on-site demonstration, group practice, discussion and reflection	4
	4.2 Mobile cabin hospital safety assurance	Theoretical instruction, on-site demonstration, discussion and reflection	2
5. Recovery	5.1 Post-disaster psychological relief	Theoretical instruction, video demonstration, on-site demonstration, scenario exercise	4
	5.2 Post-disaster health education		
6. Communication	6.1 Effective communication skills for disaster relief	Theoretical instruction, video demonstration, scenario exercise	2
7. Legal and ethics	7.1 Disaster-related legal ethics	Theoretical instruction, case discussion	1
8. Disaster management	8.1 Mobile cabin hospital emergency care management	Tabletop exercise, role playing, comprehensive exercise, discussion and reflection	4

## 4 Discussion

### 4.1 Importance of training program construction

Previous research has consistently demonstrated that newly graduated nurses often exhibit inadequate preparedness for disaster response, whereas nurses who have received disaster-specific training report significantly higher levels of readiness (7, 22–27). As a result, the development of disaster nursing competency is recognized as a critical factor in improving overall disaster response capability among nurses (28). Undergraduate nursing students are at a formative stage of professional development, during which their attitudes and conceptual understanding are particularly receptive to targeted educational interventions. This stage is considered the optimal period for integrating disaster nursing education into the curriculum (16, 27, 28).

The training program developed in the present study differs from conventional disaster training models that primarily emphasize preparedness and emergency response. Instead, this program adopts a structured, week-long format aligned with the complete disaster management cycle, including mitigation, preparedness, response, and recovery phases. Unlike the NDLS curriculum, this program not only emphasizes disaster triage and treatment protocols but also uniquely incorporates a “cabin hospital construction and operation” module—a feature rarely seen in existing international training systems. While the EPR framework focuses on cross-institutional collaboration, this program highlights efficient operations and team coordination within single-institutional settings with limited resources. As a result, it offers distinctive advantages in global disaster care education. Moreover, it incorporates practical clinical training in real-world mobile cabin hospital environments. Engaging nursing students early in such experiential learning is expected to enhance their awareness, improve disaster-related competencies, and increase their willingness to participate in future relief efforts. This approach may contribute to the development of a more resilient clinical reserve workforce capable of responding effectively in a variety of disaster contexts.

### 4.2 Scientific validity and reliability analysis of training program setup

At present, disaster nursing practical training programs tailored to undergraduate nursing interns in China remain in the exploratory phase, lacking a unified or standardized structure (4). In response to this gap, the present study utilized the ICN disaster competency framework and relevant literature as foundational references. Through the application of the Delphi, comprehensive expert feedback was gathered to guide the design and validation of the proposed Disaster Nursing Practice Week program. The training model was constructed through a rigorous and systematic process, ensuring both scientific validity and structural feasibility. The credibility of this methodological approach is supported by high expert response rates, elevated levels of expert authority, and a strong degree of consensus achieved across consultation rounds.

#### 4.2.1 Representativeness of consultation experts

The expert panel included 15 experts from Beijing, Fujian, Sichuan, and Yunnan. Participants ranged in age from 35 to 56 years, with 20% under the age of 40, ensuring a reasonable age stratification. This allowed for the integration of senior experts’ in-depth professional experience and the innovative perspectives of younger professionals. Educational qualifications were generally high, with 93.3% of experts holding a master’s degree or above, indicating reliable academic and clinical backgrounds. The panel comprised experts from tertiary hospitals and academic institutions, representing diverse fields such as nursing education, nursing management, clinical nursing, emergency medicine, and disaster medical rescue. Furthermore, 11 experts had disaster relief experience (73.33%), 10 held associate senior-level or higher titles (66.67%), and 13 had over 15 years of professional experience (86.66%). This combination of geographic diversity, academic and clinical expertise, and appropriate stratification enhanced the representativeness and comprehensiveness of the expert panel.

#### 4.2.2 Enthusiasm and authority of consultation experts

High response rates in Delphi consultations are indicative of strong engagement and alignment between the study topic and participants’ expertise (29). In the present study, both rounds of consultation achieved a 100% response rate, with 14 experts submitting detailed opinions across the two rounds. These results reflect a high degree of participation and interest. Expert authority was further assessed through self-reported familiarity and the rational basis for indicator evaluation. The calculated authority coefficient was 0.93, exceeding the accepted threshold of 0.80, thereby indicating that the experts possessed high levels of expertise and familiarity with disaster nursing, contributing to the credibility and reliability of the consultation findings.

#### 4.2.3 High degree of consensus among consultation experts

Across the two rounds of expert consultation, the indicators in the training program consistently met established thresholds: mean importance scores ≥ 4.0, full score rate ≥ 20%, selection rate ≥ 80%, and coefficient of variation ≤ 0.20 (29). Based on expert feedback and internal discussions, the training modules, instructional content, duration, and teaching methods were finalized. The Kendall’s W coefficient reached 0.300 following the second round, aligning with the recommended coordination coefficient range of 0.3 to 0.5, with a *p* value <0.001 indicating statistical significance (30). These results indicate a high degree of consensus and coordination among expert opinions, supporting the reliability and validity of the finalized training program.

### 4.3 Rationality analysis of training program setup

#### 4.3.1 Alignment with the eight core competencies of disaster nursing

The ICN and the World Health Organization (WHO) have jointly emphasized that nurses must be equipped with foundational knowledge and skills in disaster management in order to fulfill their roles effectively across all phases of disaster response (1). In 2009, the ICN and WHO released the disaster nursing competency framework, which was updated in 2019 to define core disaster nursing competencies tailored to nurses at varying levels of responsibility (9). These competencies encompass eight key domains: preparation and planning, communication, disaster management, safety assurance, assessment, intervention, recovery, and legal and ethical responsibilities. The development of these capabilities establishes the foundation for nursing personnel to assume effective roles during disasters. Level I competencies, applicable to registered nurses, include the ability to utilize basic first aid and professional nursing skills in disaster response scenarios (9, 11). As undergraduate nursing students represent a key reserve resource for disaster relief, the training program in this study was designed in accordance with the competencies outlined for registered nurses in the ICN framework. The primary indicators directly apply the eight core competencies of disaster nursing, closely adjusting the eight indicators according to the entire disaster management process, ultimately resulting in 47 secondary indicators. The program integrates competencies spanning pre-disaster preparation and planning, intervention during disasters, and post-disaster recovery, thereby facilitating comprehensive enhancement of disaster nursing competency among nursing interns. Compared to classic international models such as the National Disaster Life Support (NDLS) curriculum from the United States, the training program designed in this study also emphasizes core competencies such as ‘triage and on-site rescue,’ reflecting global consensus. However, the uniqueness of this training program lies in using mobile cabin hospitals as the core teaching scenario, designing a complete and immersive training process, providing a more aligned with real combat and systematic operational experience.

#### 4.3.2 Integration with mobile cabin hospital training contexts

Disaster events are inherently unpredictable and are characterized by abrupt onset, high risk, and operational complexity, making real-world disaster scenario training impractical. As a result, simulation-based training has emerged as a highly effective educational strategy (22, 31–34). Simulation enables learners to develop skills and practice response techniques in controlled environments without endangering actual patients, while also improving assessment proficiency (22). Evidence from multiple studies indicates that simulated disaster environments enhance understanding of nursing roles, promote interdisciplinary collaboration, and strengthen team-based disaster mitigation and response efforts (22, 31–35). In the present training program, mobile cabin hospitals are used as the central context for simulation-based instruction. The curriculum includes pre-deployment preparation, facility setup, patient assessment, safety protocols, and management of the disaster response continuum. Compared to previous training, the training based on mobile cabin hospital mainly includes the following new aspects: First, it covers the preparation before the construction of a mobile cabin hospital and the setup process, which was not included in previous courses. This primarily trains medical staff’s organizational and planning abilities, as well as the process of building a hospital upon arriving at a disaster site, to familiarize them with the various functional units of the mobile hospital. Second, it focuses on the safety assurance of mobile field hospitals, which differs from routine safety assessments at disaster sites by adding management of the hospital environment, electricity, oxygen usage, etc. Third, it involves conducting various treatment techniques within mobile cabin hospitals, as well as psychological care. Third, it includes various treatment techniques conducted within mobile cabin hospitals, as well as psychological care and early rehabilitation care for patients after treatment. These components create immersive large-scale scenarios that allow students to experience and participate in each phase of disaster relief, thereby improving their perception and operational capabilities in disaster contexts.

#### 4.3.3 Consideration of the specific demands of disaster relief

The training program was designed with explicit attention to the unique challenges of disaster relief. Given the urgent, unpredictable nature of disaster events, the curriculum includes modules on disaster knowledge, preparation of personal emergency equipment, and mobile cabin hospital readiness. To address the clinical challenges associated with mass casualty events and the need to care for vulnerable populations—including older adults, children, pregnant and postpartum women, bereaved individuals, and persons with disabilities—the program incorporates targeted modules on injury assessment, medical interventions, and population-specific nursing care. The program also incorporates components that address infectious disease containment, psychological support for both responders and victims, post-disaster health education, and ethical and legal challenges encountered in disaster scenarios. Additionally, recognizing the importance of interprofessional coordination during disaster response, the training also includes dedicated content on communication skills to enhance collaborative response effectiveness.

#### 4.3.4 Alignment with the practical focus of clinical training

Currently, disaster nursing training in our country mainly targets in-school students, with relevant chapters added only to critical care nursing courses, or conducted sporadically as elective courses, without any clinical practice courses. A well-documented limitation in disaster nursing education is the gap between theoretical instruction and practical application in clinical settings (28). To address this gap, the current program focuses on the practical training needs of undergraduate nursing interns. The curriculum emphasizes hands-on participation in mobile cabin hospital deployment, triage procedures, trauma assessment, and essential clinical interventions such as hemostasis, bandaging, immobilization, transport, transfer, and cardiopulmonary resuscitation. Technical instruction includes the use of Level II personal protective equipment, rapid psychological assessment techniques, self-regulation strategies, and communication techniques. Through direct engagement with these practical tasks, nursing interns are better equipped to translate theoretical knowledge into practical competencies applicable in real-world disaster contexts.

### 4.4 Multi-modal training methods enhance instructional effectiveness

The number of disaster nursing training programs has steadily increased in recent years. While traditional training methods have relied heavily on didactic instruction, recent approaches have incorporated group discussion, action learning, problem-based learning, virtual platforms, disaster scenario simulations, tabletop exercises, and flipped classroom formats (22, 31–40). Although individual instructional methods can yield positive outcomes, the multifaceted nature of disaster nursing and the diverse learning preferences among students limit the effectiveness of single-mode approaches (23). Ngo et al. advocated for multi-modal, integrated training strategies as an effective approach to disaster education (41). Moreover, research indicates that no single instructional format has demonstrated superiority across all competency domains (22). Consequently, multi-modal teaching methods are recommended to optimize student engagement and competency acquisition. These methods have been shown to enhance performance in critical areas such as disaster perception, triage, clinical skills, crisis management, and problem-solving. For example, training conducted in mobile cabin hospital contexts has been demonstrated to improve situational awareness, while virtual reality and tabletop simulations have demonstrated effectiveness in improving disaster-related skills and perceptions (25, 42–45). Additional studies have reported that the use of disaster scene imagery, video demonstrations, case analyses, and personal narratives can increase nurses’ awareness of the need for continuous professional development, and enhance their confidence and motivation for participating in relief efforts (44). Therefore, the present training program incorporates a variety of instructional methods tailored to specific content domains, aiming to improve learning outcomes, increase student interest, and strengthen the acquisition of core disaster nursing competencies.

### 4.5 Research limitations

This study also has certain limitations. Firstly, the scheme established by this study heavily relies on the opinions of the expert panel. Although we invited experienced experts in the field, the majority of the panel members come from the same domestic healthcare system, which has geographical and institutional limitations. This may result in the scheme being more tailored to the specific operational model and cultural background of our country’s makeshift hospitals, and its general applicability, especially in different countries or healthcare systems, still needs further evaluation. Secondly, the current study has only completed the theoretical construction and preliminary validation of the scheme, without large-scale empirical application and effectiveness verification in real mobile makeshift hospitals. Therefore, the actual effectiveness of the scheme in improving team collaboration and patient outcomes still needs to be confirmed by subsequent empirical research. Finally, when constructing the scheme, this study mainly considered the typical static workflow of makeshift hospitals and did not fully incorporate their highly dynamic operational characteristics, such as sudden fluctuations in the number of patients, temporary supplementation of medical resources, dynamic expansion or reduction of cabin space, etc. These dynamic factors impose requirements for the flexibility and adaptability of the training scheme, which need to be key considerations in future research and updates to the scheme.

## 5 Conclusion

The present study adopted the revised *Disaster Nursing Core Competencies* released by the ICN in 2019 as the guiding theoretical framework and integrated these competencies with the operational context of mobile cabin hospitals. Through a comprehensive literature review and expert consultation process, a structured “disaster practice week” training program was developed for undergraduate nursing interns. The finalized program comprises 8 primary indicators, 14 secondary indicators, and 47 tertiary indicators. It incorporates clearly defined training content, instructional methods, and a designated schedule of training hours. The result is a comprehensive, practical, and adaptable model tailored to the specific demands of disaster nursing education. This program offers a standardized framework for the future implementation of disaster nursing training among nursing interns and holds significant potential for strengthening the preparedness and professional development of future disaster nursing personnel. In the future, we will conduct small-scale pilot studies through simulation exercises, using a pre-and-post comparison design, to preliminarily test the effectiveness and feasibility of the training program.

## Data Availability

The original contributions presented in the study are included in the article/supplementary material, further inquiries can be directed to the corresponding authors.
